# Acute kidney injury, secondary to valacyclovir use during pregnancy for the prevention of fetal cytomegalovirus infection

**DOI:** 10.1002/ijgo.71021

**Published:** 2026-04-18

**Authors:** Jason Brafman, Yechiel Schlesinger, Moshe Barg, Shmuel Benenson

**Affiliations:** ^1^ Department of Internal Medicine Shaare‐Zedek Medical Center Jerusalem Israel; ^2^ Department of Pediatrics Shaare‐Zedek Medical Center Jerusalem Israel; ^3^ Faculty of Medicine The Hebrew University of Jerusalem Jerusalem Israel; ^4^ Department of Obstetrics & Gynecology Shaare‐Zedek Medical Center Jerusalem Israel; ^5^ Unit for Infection Prevention and Control Shaare‐Zedek Medical Center Jerusalem Israel

**Keywords:** acute kidney injury, cytomegalovirus, fetal CMV infection, valacyclovir

## Abstract

High dose valacyclovir treatment of pregnant women has become a common treatment for primary CMV infection, to prevent vertical transmission to the fetus. We present a case of a healthy young pregnant female with primary CMV infection during the first trimester who was diagnosed with acute kidney injury secondary to valacyclovir treatment. This case serves as a reminder to clinicians that prescription of high dose valacyclovir, to prevent vertical transmission of CMV to the fetus, carries a risk of approximately 1% of AKI.

Cytomegalovirus (CMV) is a ubiquitous virus that can cause mononucleosis‐like illness in children and adults. In pregnant women, it poses the danger of affecting the fetus, especially in the case of a primary infection acquired early in pregnancy. Symptoms of fetal CMV infection contracted in utero, also known as congenital CMV, include diverse neonatal manifestations such as sensorineural hearing loss, seizures, “blueberry muffin” rash, and chorioretinitis.

Valacyclovir administration to avert vertical transmission of CMV infection has increased in recent years, since the randomized controlled trial by Shahar‐Nissan et al. in 2020.^1^ In this study, early treatment of infected women with valacyclovir (until performance of amniocentesis at 21 weeks of pregnancy) exhibited a significant reduction in the vertical transmission rate compared with placebo. Acute kidney injury (AKI) has been previously documented to occur due to oral acyclovir.^2^, ^3^, ^4^ Valacyclovir, a prodrug of acyclovir, has greater bioavailability than acyclovir. Its higher serum levels further aggravate the threat of AKI, and more so when high‐dose valacyclovir (e.g. 4 g twice daily) is prescribed, which is used to minimize maternal to fetal transmission of CMV. This dose is significantly higher than the usual dose for other indications (1–2 g twice daily).^5^ However, in that study there was no known occurrence of AKI among the 50 pregnant women that were randomized to receive valacyclovir.

We present a case of a healthy 24‐year‐old woman with normal kidney function tests, who had a negative screening test for CMV serology at Week 4 of pregnancy. At Week 13, she had seroconversion to positive anti‐CMV IgM and IgG titers with low avidity, which indicated primary CMV infection during the first trimester. Treatment with oral valacyclovir 4 g twice daily was initiated. After beginning valacyclovir, the patient began experiencing nausea and was referred for repeat laboratory evaluations, including kidney and liver function tests. The kidney function tests showed a serum creatinine of 4.2 mg/dL (normal range 0.66–1.25 mg/dL) and blood urea nitrogen (BUN) of 36 mg/dL (normal range 9–20 mg/dL); complete blood count and liver function tests were within normal limits.

The patient was diagnosed with acute kidney injury secondary to valacyclovir. Treatment with valacyclovir was discontinued. The patient was hospitalized for 5 days to receive treatment with intravenous fluids and kidney function monitoring. She did not require hemodialysis. The creatinine and BUN gradually improved (2.06 and 16 mg/dL, respectively) and the patient was discharged to repeat kidney function tests in 1 week to ensure complete resolution of AKI.

In the 2023 meta‐analysis of d'Antonio et al.,^6^ which included eight studies, women treated with valacyclovir had a significantly lower risk of congenital CMV infection compared with those not receiving valacyclovir (odds ratio 0.37, 95% confidence interval 0.21–0.64). In their analysis, only two out of 210 women treated with valacyclovir developed AKI (0.95%) (one described in Egloff et al.^7^ and one in Faure‐Bardon et al.^8^). Both women had a recovery of kidney function upon cessation of valacyclovir, as in our case.

Our case, as well as the cases documented by Egloff et al.^7^ and Faure‐Bardon et al.,^8^ serve as a reminder to clinicians that prescription of high‐dose valacyclovir to prevent vertical transmission of CMV to the fetus, carries a risk of approximately 1% of AKI. This potential complication demands preventative measures and monitoring to mitigate the risk. Specifically, we suggest instructing the treated woman to drink at least 2 L of water daily and to consider obtaining weekly creatinine and BUN serum levels until the conclusion of valacyclovir treatment.

Although the incidence of AKI due to valacyclovir is around 1%,^6^ as more pregnant women receive valacyclovir for CMV infections to avoid transmission of the infection to their fetus, the prevalence of AKI will increase accordingly. This threat to maternal kidney function is reversible upon termination of valacyclovir, if noticed early enough, and these women should be monitored carefully.

The Ethics Committee of Shaare‐Zedek Medical Center exempted us from the obligation to request its approval to conduct the above study. Informed consent was obtained from the patient for publication of the clinical data.

## AUTHOR CONTRIBUTIONS

JB and SB initiated the study, participated in data collection, and wrote the manuscript. They are accountable for the accuracy and integrity of the work. YS and MB participated in the data collection, data analysis, and writing of the manuscript and have approved the publication of this version.

## FUNDING INFORMATION

The authors have nothing to report.

## CONFLICT OF INTEREST STATEMENT

The authors have no conflicts of interest.

## Data Availability

Data sharing is not applicable to this article as no new data were created or analyzed in this study.
